# Controlled human infectious models, a path forward in uncovering immunological correlates of protection: Lessons from enteric fevers studies

**DOI:** 10.3389/fmicb.2022.983403

**Published:** 2022-09-20

**Authors:** Marcelo B. Sztein, Jayaum S. Booth

**Affiliations:** ^1^Center for Vaccine Development and Global Health, University of Maryland School of Medicine, Baltimore, MD, United States; ^2^Department of Pediatrics, University of Maryland School of Medicine, Baltimore, MD, United States; ^3^Department of Medicine, University of Maryland School of Medicine, Baltimore, MD, United States

**Keywords:** typhoid, correlates of protection, immunity, challenge human infection model, vaccine, enteric diseases

## Abstract

Enteric infectious diseases account for more than a billion disease episodes yearly worldwide resulting in approximately 2 million deaths, with children under 5 years old and the elderly being disproportionally affected. Enteric pathogens comprise viruses, parasites, and bacteria; the latter including pathogens such as *Salmonella* [typhoidal (TS) and non-typhoidal (nTS)], cholera, *Shigella* and multiple pathotypes of *Escherichia coli* (*E. coli*). In addition, multi-drug resistant and extensively drug-resistant (XDR) strains (e.g., *S.* Typhi H58 strain) of enteric bacteria are emerging; thus, renewed efforts to tackle enteric diseases are required. Many of these entero-pathogens could be controlled by oral or parenteral vaccines; however, development of new, effective vaccines has been hampered by lack of known immunological correlates of protection (CoP) and limited knowledge of the factors contributing to protective responses. To fully comprehend the human response to enteric infections, an invaluable tool that has recently re-emerged is the use of controlled human infection models (CHIMs) in which participants are challenged with virulent wild-type (wt) organisms. CHIMs have the potential to uncover immune mechanisms and identify CoP to enteric pathogens, as well as to evaluate the efficacy of therapeutics and vaccines in humans. CHIMs have been used to provide invaluable insights in the pathogenesis, host-pathogen interaction and evaluation of vaccines. Recently, several Oxford typhoid CHIM studies have been performed to assess the role of multiple cell types (B cells, CD8+ T, T_regs_, MAIT, Monocytes and DC) during *S*. Typhi infection. One of the key messages that emerged from these studies is that baseline antigen-specific responses are important in that they can correlate with clinical outcomes. Additionally, volunteers who develop typhoid disease (TD) exhibit higher levels and more activated cell types (e.g., DC and monocytes) which are nevertheless defective in discrete signaling pathways. Future critical aspects of this research will involve the study of immune responses to enteric infections at the site of entry, i.e., the intestinal mucosa. This review will describe our current knowledge of immunity to enteric fevers *caused byS.* Typhi and *S.* Paratyphi A, with emphasis on the contributions of CHIMs to uncover the complex immunological responses to these organisms and provide insights into the determinants of protective immunity.

## Introduction

Vaccination is an effective strategy to prevent infectious diseases. Vaccines effectiveness has been demonstrated in many settings with remarkable success. Notable examples include the eradication of smallpox and the near eradication of polio (Minor, 2015). However, in spite of remarkable advances in our understanding of the mechanisms of immune responses, the development of new vaccines remains somewhat empirical, slow, and is hindered by multiple factors including lack of animal models for human restricted diseases, lack of known immunological correlates of disease protection and lack of understanding of host-pathogen interaction (especially in human hosts) for most infectious diseases including enteric diseases (Levine and Sztein, 2004). These data will be crucial to advance the development of new generation vaccines and to improve on moderately effective vaccines currently in use.

Animal models have been used extensively to elucidate the correlates of protection (CoP) and protective immunity to various infectious enteric pathogens (Cai et al., 2010; Pore et al., 2011; Mayr et al., 2012). While these studies have yielded enormous insights into the pathogen-host interactions and immunity, significant differences in immune responses to challenge have been observed between animal models and humans (Mestas and Hughes, 2004). These differences have been attributed to key differences in leukocyte subsets, pattern recognition receptors [PRR; e.g., Toll like receptors (TLR)] and in the development, activation, and responses of both the innate and adaptive immune system (Mestas and Hughes, 2004). In addition, many pathogens are human restricted (e.g., *S*. Typhi) and there is no reliable animal model that can faithfully recapitulate the disease. Therefore, to address these issues, we are largely dependent on human studies. Most human studies make use of samples obtained from peripheral blood for obvious reasons (practical and ethical) to probe the immune response following vaccination or infection. Extensive data sets are available on the generated immunity elicited after enteric pathogen infection in the field or following vaccination with candidate enteric vaccines. However, the immunological status before wild-type infection is usually unclear, and its potential role on clinical outcome undefined.

Recently, controlled human infection models (CHIMs) have received increased attention. CHIM involves the intentional infection of healthy, consenting volunteers with a well-characterized strain of an infectious pathogen in order to understand human diseases by attempting to uncover CoP, host-pathogen interactions and to evaluate the efficacy of new vaccines and therapeutics. It is important to point out that CHIM studies are conducted only when the risk to the participants and the community is deemed to be reasonable compared to the study benefits including benefits to the volunteers (if any) and the knowledge gained (Weijer and Miller, 2004). Thus, the risks for CHIM studies should be clearly and accurately defined. Pathogens that are used in bacterial CHIM studies should be extensively characterized to ensure that either they are highly sensitive to antibiotic treatment or are attenuated strains unable to disseminate to other people or cause disease in the participants (Weijer and Miller, 2004). Furthermore, appropriate laboratory and animal studies whenever possible should be performed and completed before human challenges are initiated. These data must have been reported and available in systematic reviews of the literature (Weijer and Miller, 2004). An important safety component of CHIMs to minimize the risk of the volunteers to develop severe complication is the administration of curative treatments at the onset or diagnosis of disease. For example, in malaria CHIM studies, only treatment-sensitive malaria strains are used resulting in the absence of severe or life-threatening malaria in any of the volunteers challenged up to date (Church et al., 1997; Epstein et al., 2007). Additionally, CHIM studies are important in investigating pathogens for which there is no availability of adequate animal models that faithfully reproduce human disease, or in which infections are rare. Also, during emerging epidemics and pandemics, CHIMs, if possible, have the advantage that they may contribute to rapidly collecting data about vaccine candidates. Furthermore, CHIM studies have reduced both the time and costs involved in vaccine development and have helped curb the risks associated with vaccine development.

CHIMs have been carried out for over 20 different pathogens and have been used to understand disease pathogenesis and evaluate candidate vaccines. For example, between 1952 and 1974, Drs. Theodore Woodward and Richard B. Hornick conducted extensive typhoid challenge studies at the University of Maryland which provided key insights into typhoid pathogenesis and the efficacy of the live attenuated typhoid vaccine Ty21a (Hornick et al., 1970, 2007; Gilman et al., 1977). Using the Quailes *S.* Typhi strain used in the Maryland challenge trials, Dr. Pollard’s Oxford Vaccine Group recently initiated experimental typhoid challenge studies in community participants (Waddington et al., 2014b; Darton et al., 2015). In addition to enteric fevers, CHIMs are currently being used to assess preliminary efficacy of prototype vaccine candidates for several intestinal pathogens, including *Shigella*, *Campylobacter*, enterotoxigenic *Escherichia coli* (ETEC) and norovirus (Kirkpatrick et al., 2013; Brubaker et al., 2021; Talaat et al., 2021). These CHIMs are likely to speed up the selection of promising vaccine candidates and contribute to our knowledge of disease pathogenesis and immunological CoP. In this review, we will focus on the lessons learn from CHIMs using the examples of the typhoid and paratyphoid human challenge models, particularly in terms of the evaluation of vaccine candidates, immunity and the potential CoP emanating from these studies using modern technologies.

## Evaluation of typhoid vaccine candidates using CHIM

The evaluation of potential new vaccine candidates is usually a lengthy, laborious and expensive process which is complicated by numerous factors including, among others, human host-restriction, lack of clear immunological CoP, and suboptimal diagnostics leading to uncertainties in evaluating vaccine impact in protection. CHIM can be used for the acceleration of vaccines candidate selection since it can overcome some of these limitations by providing a direct measurement of vaccine efficacy, clear diagnosis of disease in a control environment and provide specimens and data to assist in the identification of immunological CoP. Remarkably, the establishment of a CHIM for typhoid fever was pioneered by Drs. Theodore Woodward and Richard B. Hornick at the University of Maryland in the 1950s, 1960s, and 1970s to assess typhoid vaccines using the Quailes strain (wild type, Vi-expressing *S*. Typhi) as the challenge organism (Hornick et al., 1970, 2007; Gilman et al., 1977; Woodward, 1980). These studies investigated six different vaccines and contributed a vast amount of information regarding typhoid pathogenesis reviewed in (Gilman et al., 1977; Levine et al., 2001; Hornick et al., 2007; Waddington et al., 2014b). Interestingly, these trials also led to the development of Ty21a, an oral live attenuated typhoid vaccine, which shows around 87% efficacy after multiple doses (Gilman et al., 1977). Subsequently Ty21a underwent large scale field trials based on these promising results and is now one of the licensed vaccines for typhoid fever (Levine et al., 1987). Furthermore, four CHIM studies were performed with streptomycin dependent *S*. Typhi as an oral attenuated vaccine (Levine et al., 1976). The authors reported that freshly harvested vaccine was highly protective (66–78% efficacy), but not the lyophilized formulation (Levine et al., 1976). Thus, the authors used a CHIM to demonstrate that a streptomycin *S*. Typhi candidate vaccine retains its protective properties when used fresh, but not when lyophilized, thus limiting the use of this candidate vaccine (Levine et al., 1976). Interestingly, a re-analysis of the clinical data generated in CHIM studies between 1959 and 1970 was conducted by Dr. Hornick at the University of Maryland School of Medicine to examine the relationship between challenge dose and severity of disease (Glynn et al., 1995). The authors found that using a stringent definition of illness, there was no association between dose of challenge and severity of disease. However, when the criteria were relaxed, some association was observed between dose and severity (Glynn et al., 1995). Of note, although these CHIMs were used successfully, the human challenge model was halted due to ethical concerns at the time.

Until recently, licensed typhoid vaccines were moderately efficacious needing multiple doses in the case of Ty21a, and more importantly they were unsuitable for use in children less than 2 years old. In 2014, the Oxford Vaccine Group (University of Oxford) re-established the typhoid CHIM in which immunologically naïve, consenting volunteers ingested wild-type (wt) *S*. Typhi (Quailes strain) in an ambulatory setting (Darton et al., 2014; Waddington et al., 2014a). Using this CHIM, these investigators evaluated the protective efficacy of a single dose of an oral vaccine candidate, M01ZH09 and compared it to Ty21a (Darton et al., 2016). The authors found that the vaccine efficacy for one dose of M01ZH09 was 13% as compared to 35% for 3 doses of Ty21a in this model using the Oxford typhoid diagnosis definition (either fever 38^°^C for >12 h, with or without isolation of *S.* Typhi from blood, or isolation of *S.* Typhi from blood -bacteremia- even if silent without any clinical signs or symptoms of illness). Thus, one dose of M01ZH09 did not significantly protect volunteers after challenge with *wt S*. Typhi in this CHIM. As a result, the M01ZH09 vaccine was not further evaluated in clinical studies.

Of importance, *S*. Typhi conjugate vaccines have long been sought and have been initially generated using the capsular polysaccharide of *Salmonella* Typhi, Vi, linked to recombinant aeruginosa exotoxin A (rEPA; Lin et al., 2001). This vaccine candidate was evaluated in the field for its safety, immunogenicity and efficacy in children 2–5 years old in Vietnam (Lin et al., 2001). The authors reported that the conjugate typhoid vaccine was safe, immunogenic and has a 90% efficacy in children 2–5 years old (Lin et al., 2001). This study and others paved the way for the new generation of conjugated typhoid vaccine such as Vi-TT (tetanus toxoid). Using the typhoid CHIM, Vi-conjugated vaccine (Vi-TT, tetanus toxoid), as well as the licensed Vi polysaccharide vaccine (Vi-PS) were evaluated for vaccine efficacy (Jin et al., 2017). In this study, the authors demonstrated that Vi-TT had a vaccine efficacy of 54.6% with a seroconversion rate of 100% while Vi-PS has 52% vaccine efficacy with 88.6% seroconversion rate (Jin et al., 2017). They concluded that Vi-TT is a highly immunogenic vaccine that could potentially reduce the burden of typhoid fever. In fact, this CHIM provided key data to support the World Health Organization (WHO) Strategic Advisory Group of Experts on Immunization (SAGE) recommendation to use typhoid conjugate vaccines such as Vi-TT (Typbar-TCV) in children from 6-month-old in typhoid endemic regions. Fields trials have since been underway in countries with high burden of disease and/or high rates of antimicrobial resistance (Neuzil et al., 2020). Recently, the result of an efficacy of Vi-TT vaccine in Nepal was reported (Shakya et al., 2021). The field trial enrolled 20,019 children aged 9 months to 16 years who received a single dose of TCV or capsular group A meningococcal conjugate vaccine (MenA). The group reported a protective efficacy of 79% against blood culture confirmed typhoid fever at 2 years (Shakya et al., 2021). Similar results were observed in children participating in a large field study in Bangladesh (Qadri et al., 2021). Thus, the use of CHIMs have a real-world impact on vaccine evaluation and introduction of vaccines to endemic regions. CHIMs are clearly an invaluable tool in a wider toolkit that includes field trials and studies directed primarily to explore the immunogenicity of novel vaccine candidates (Mohan et al., 2015). Importantly, this, and other CHIM models, are uniquely well suited to study in great detail the immunological responses present before, as well as during acute infection and recovery phases, to enable the identification of immunological CoP.

## Evaluation of enteric vaccines other than *Salmonella* using CHIMs

CHIM studies have been used to evaluate numerous enteric vaccines and to study the pathogenesis of enteric pathogens. In this section, because of space limitations, it is not possible to discuss all the enteric pathogens that have been evaluated using CHIMs. However, we will briefly discuss some of the most important non-*Salmonella* enteric CHIM studies and the salient findings emanating from these studies. Early *Shigella* CHIMs were used to evaluate potential *Shigella* vaccine candidates, but there were limitations in terms of low and variable attack rates because of the use of skim milk as a delivery vehicle. In a pivotal study, Kotloff et al., showed that the use of bicarbonate buffer instead of skim milk in a *Shigella* CHIM study, allowed for a more reproducible CHIM and higher attack rates (Kotloff et al., 1995). This technique was safe, reproducible and valid for the selection of *Shigella* vaccines and other agents (Kotloff et al., 1995). This method has since been used in other *Shigella* and non-*Shigella* studies. CHIM standardization is necessary in order to compare potential vaccine candidates across institutions and over time. Thus, in November 2017 at the Vaccine Against *Shigella* and ETEC (VASE), a *Shigella* CHIM workshop was organized which addressed multiple issues related to the CHIM studies. These issues were discussed, and consensus reports were published on the most critical component of CHIM including (i) Introduction and overview (MacLennan et al., 2019a), (ii) Conduct of Studies (Porter et al., 2017; Talaat et al., 2019), (iii) Clinical endpoints (MacLennan et al., 2019b; Porter et al., 2019), and (iv) Immunological assays (Kaminski et al., 2019). These consensus reports constitute a framework to establish common procedures for the advancement of *Shigella* vaccine development, licensure, and distribution. One of the improvements of the *Shigella* CHIM focuses on the standardization of the model through the use of lyophilized *Shigella* challenge strains rather than plate grown inoculum preparations. Using lyophilized *S*. *sonnei* 53G strain, Frenck et al., established a *Shigella* CHIM and showed that a dose in the range of 1,500 to 2,000 CFU of 53G was adequate for future challenge studies (Frenck et al., 2020). This study is very important in the path forward to standardize *Shigella* CHIM. The CoP for *Shigellosis* have yet to be defined and two recent *Shigella* CHIM studies have examined the immune responses in order to define the CoP (Clarkson et al., 2020, 2021). In the first study, the authors reported that there was an association between higher level of *Shigella* LPS-specific serum IgA and memory B cell IgA responses at baseline with reduced risk of disease using the lyophilized *S. sonnei* 2G as challenge strain (Clarkson et al., 2020). In the second study, they used two challenge strains, *S. sonnei* and *S. flexneri* 2a and showed that the two different *Shigella* serotypes induces distinct innate and adaptive immune profiles post-oral challenge (Clarkson et al., 2021). These data demonstrate that the immune mechanisms required for protective immunity may be dependent on the Shigella serotype which would impact significantly on Shigella vaccine development. CHIM studies for another enteric pathogen, *V. cholerae*, were performed to assess the protective efficacy of a live oral cholera vaccine, CVD103-HgR. Using the cholera CHIM, Tacket et al., demonstrated that CVD103-HgR provided significant protection against classical Inaba strain 569B and El Tor *Vibrio Cholerae* O1 following vaccination with CVD103-HgR (Tacket et al., 1992). This protection was evident as early as 8 days and persisted for at least 6 months. Vaccine efficacy was 90.3 and 79.5% at 10 days and 3 months post vaccination (Tacket et al., 1992). The findings from this efficacy study combined with immunogenicity and safety data and a more recent cholera CHIM study, played an instrumental role in the licensing of the CVD103-HgR vaccine by the FDA (Tacket et al., 1992; Mosley et al., 2017). Further cholera CHIM studies established: (i) a model of South American cholera used to predict field vaccine efficacy in a population with high prevalence of blood group antigen O (Tacket et al., 1995) and (ii) factors influencing secondary vibriocidal immunes responses (Losonsky et al., 1996). Moreover, CHIM studies were performed to assess the effect of prior enteropathogenic *Escherichia coli* (EPEC) infections on protective immunity following homologous and heterologous rechallenge (Donnenberg et al., 1998). The authors showed that following homologous challenge, prior EPEC infections can reduced disease severity (Donnenberg et al., 1998). In fact, disease severity was inversely correlated with the level of pre-challenge serum immunoglobulin G against the 0127 LPS (Donnenberg et al., 1998). Furthermore, an *E. coli* CHIM study was performed to examine the diarrheagenic potential of two new strains of diffusely adherent (DA) *Escherichia coli*. The authors could not show the pathogenic potential of DA *E. coli* in their CHIM model (Tacket et al., 1990) demonstrating the potential of CHIM studies to investigate pathogenesis. Recently, at VASE, a workshop was set up to address some of major issues about ETEC CHIM including standardization, volunteer selection and screening and strain selection. The main recommendations were: (i) further standardization of ETEC CHIM, (ii) additional challenge strains to be developed, (iii) volunteer screening and selection to be more stringent (Hanevik et al., 2019). Altogether, these recommendations may maximize the contribution of CHIM to our understanding of ETEC pathogenesis and development of vaccines.

Finally, CHIM studies can also be used to study enteric pathogens other than bacteria, e.g., the Giardia parasite that causes the diarrheal disease giardiasis (Nash et al., 1987). A giardia lamblia CHIM was used to assess the pathogenicity of two distinct human isolates (GS/M and Isr; Nash et al., 1987). The authors showed that the pathogenicity of Giardia infections in humans varies according to the Giardia strain used in the challenge (Nash et al., 1987). All these examples of enteric CHIM demonstrate their use and importance in further our understanding of enteric diseases.

## CHIM: Understanding immunity in infectious diseases (e.g., *Salmonella*)

The ultimate goal of vaccination is to elicit short- and long-term protective immunity against infectious diseases. However, there is no universal guidance regarding protective immunity for individual vaccines. Depending on the characteristics of the infectious disease, the immunological CoP will differ; hence the immunological CoP must be identified individually for each pathogen, even closely related infectious organisms. While the use of animal models has been extensively used for this purpose, it is well known that there are significant differences between the immunity elicited in animals and humans, particularly regarding CoP. Thus, human studies are needed to confirm or identify CoP. CoP usually involve one or more markers that correlates significantly with protection following vaccination. The CoP may be either the mechanistic cause of protection (mCoP) or not be involved directly in protection but nevertheless predicts protection (non-mechanistic—nCoP; Plotkin and Gilbert, 2012). For example, serum IgG antibodies to *Shigella* LPS have been identified as a mCoP against Shigellosis (Cohen et al., 2019). Human studies utilizing peripheral blood mononuclear cells (PBMC), and more recently mucosal biopsy samples obtained from volunteers immunized with licensed (e.g., Ty21a) vaccines have contributed to our understanding of the immunity elicited by *S*. Typhi.

### Systemic and mucosal cell-mediated immunity to *S*. Typhi

*Salmonella enterica* serovar Typhi (*S*. Typhi) is a facultative, intracellular, human-restricted pathogen that causes typhoid fever, a major global health threat. The burden of *S*. Typhi infection is an estimated 26.9 million cases of typhoid fever annually resulting in approximately 217,000 deaths worldwide (Crump et al., 2004; Bhutta and Threlfall, 2009; Crump and Mintz, 2010; Levine, 2018). Following ingestion, *S*. Typhi invades the host intestinal cells (“M” and epithelial cells) and subsequently translocate to the submucosa and disseminate to the liver, spleen and other secondary lymphoid tissues, resulting in systemic illness (Levine, 2018). *S*. Typhi immunity is very complex and engage all arms of immunity including innate immunity, as well as adaptive (humoral and CMI) immunity. Most of the data generated to understand *S*. Typhi immunity used PBMC obtained from volunteers immunized with various typhoid licensed (e.g., Ty21a, Vi) and other candidate vaccines. For the last four decades, our group and others have studied extensively *S*. Typhi CMI immunity and showed that both CD4+ and CD8+ are induced to proliferate and produce a wide array of cytokines and chemokines with propensity to type 1 [T helper 1 (T_H_1) and T cytotoxic 1 (Tc1)] proinflammatory cytokines, which include, among others, interferon (IFN)-γ, tumor necrosis factor (TNF)-α, and interleukin (IL)-17 following vaccination (Sztein et al., 1994, 2014; Viret et al., 1999; Wyant et al., 1999; Salerno-Goncalves et al., 2002, 2003, 2004, 2010; McArthur and Sztein, 2012). Additionally, we have shown that elicited cytotoxic CD8+ T lymphocytes (CTL) were able to kill *S*. Typhi-infected autologous targets *via* a FAS-independent, granule-dependent pathway following vaccination with Ty21a (Sztein et al., 1995; Salerno-Goncalves et al., 2004) and the CVD 909 vaccine candidate (Wahid et al., 2007). Interestingly, multiple mechanisms seem to be involved in the killing of *S*. Typhi-infected cells and production of *S*. Typhi-specific cytokines (Salerno-Goncalves et al., 2002, 2003, 2004), including those involving classical Human Leukocyte Antigen (HLA) class-I and non-classical HLA-E molecules (Salerno-Goncalves et al., 2004). Furthermore, the duration of immunity in these vaccinated volunteers have been studied (up to 3 years) and showed that the responses were multiphasic and multifunctional (having more than one function simultaneously) which have been associated with protective immunity in multiple infectious diseases (Betts et al., 2003, 2006; Precopio et al., 2007; Qiu et al., 2012). These responses were mediated mostly by T effector memory (T_EM_) and CD45RA+ T_EM_ (T_EMRA_) cells and to a lesser extent by T central memory (T_CM_; Betts et al., 2003, 2006; Precopio et al., 2007; Qiu et al., 2012). These responses suggest that strong CD8+ T memory responses can be elicited and likely remain an integral feature of long-term immunity. Furthermore, our group have evaluated the antigen specificity of CD4+ and CD8+ T cells and reported 9 novel proteins (e.g., OmpH, OmpR, TviA, and TviE) capable of eliciting T cell responses (Salerno-Goncalves et al., 2020). The functional patterns of CD4+ and CD8+ T_M_ responses were different depending on the protein eliciting the immune response (Salerno-Goncalves et al., 2020). Taken together, these studies provided a wealth of information regarding the functional characteristics of *S.* Typhi immunity in humans. However, these human T memory (T_M_) studies have been largely limited to T cells isolated from peripheral blood, which represent only ~3% of T_M_ present in the body. The human gastrointestinal tract, the site of entry of *S.* Typhi, constitutes a major reservoir of total body lymphocytes (~60%) and represents an area of high antigenic exposure, but our understanding of the mechanisms of protection from bacterial infection is scarce, particularly with respect to immunologic events.

Recently, studies have started to unravel the local immunity in the intestine following vaccination with Ty21a. Our group have reported that human terminal ileum (TI) CD4+ T_M_ (Booth et al., 2019a) and CD8+ T_M_ (Booth et al., 2017) *S*. Typhi-specific responses were elicited following vaccination with oral Ty21a. These studies demonstrated that TI lamina propria mononuclear cells (LPMC) CD4+ and CD8+ *S.* Typhi-specific T cell responses were significantly increased, including IFN-γ and IL-17A production, cytotoxic T cells (CTL), and multifunctional (MF) antigen-specific cytokine-producing LPMC following Ty21a immunization. Initial analysis of the cell subsets in the mucosa revealed that Ty21a-immunization induced *S*. Typhi responsive LPMC CD4+ and CD8+ T cells by all major T_M_ subsets (e.g., IFN-γ and IL-17A in CD4+ T_EM_; IFN-γ and macrophage inflammatory protein (MIP)-1β in CD4+ T_CM_; and IL-2 in CD4+ T_EMRA_; Booth et al., 2017, 2019). In addition, we have compared *S*. Typhi-specific responses between mucosal and systemic compartments from concurrent samples (Booth et al., 2019b); review in (Booth and Toapanta, 2021) and (Toapanta et al., 2020). Our data indicated that there are important differences (quantity and quality) in the immune responses between the mucosal and systemic compartments following oral Ty21a immunization. Interestingly, studies by Pennington et al. showed that oral Ty21a immunization elicited *S*. Typhi-responsive CD4+ and CD8+ T cells obtained from human duodenum biopsies but not from T cells isolated from human colon biopsies (Pennington et al., 2016). Together, these data indicate that the mucosal immune responses elicited by oral Ty21a immunization are compartmentalized along the human intestine and that the immune responses in the local gut microenvironments differ from those in the systemic compartment.

Furthermore, we now know that protective immunity relies not only on circulating T_M_ but also on resident (non-circulating) T_M_, namely tissue resident memory T cells (T_RM_) which are abundant in peripheral tissues, especially at mucosal sites (Thome and Farber, 2015; Mueller and Mackay, 2016). T_RM_ is a relatively newly defined subset of T_M_ which is phenotypically distinct from circulating T_M_ subsets (e.g., T_CM_, T_EM_, and T_EMRA_). We have examined in detail T_RM_ and reported the effect of Ty21a vaccination on human TI CD4+ and CD8+ T_RM_ *S*. Typhi-specific responses in both the lamina propria (LP) and intraepithelial (IEL) compartments (Booth et al., 2019c, 2020). Briefly, we have demonstrated that LPMC CD4+ T_RM_ subsets contributed significantly to *S*. Typhi-specific IFN-γ, IL-17A and IL-2 responses following stimulation with *S*. Typhi-infected targets (Booth et al., 2020). Moreover, these responses differed in magnitude and characteristics between CD103+ and CD103− CD4+ T_RM_, suggesting a dichotomy in their contributions and possibly different roles in *S*. Typhi immunity (Booth et al., 2020). Interestingly, in the epithelial compartment, IEL CD103− CD4+ T_RM_ contributed significantly to *S*. Typhi specific IFN-γ, IL-17A and TNF-α while IEL CD103+ CD4+ T_RM_ contributed to IL-2 production (Booth et al., 2020). Similarly, we showed that CD8+ T_RM_ responses were primarily in T cytotoxic (Tc) 17 cells in both the lamina propria and epithelial compartments (Booth et al., 2019c). However, LPMC CD8+ CD69+ CD103– T cells subsets contributed significantly to *S*. Typhi-specific IFN-γ, IL-17A, and IL-2 responses following Ty21a immunization (Booth et al., 2019c). While taken together, these studies have considerably advanced our understanding of the pathogenesis and immunity generated by *S*. Typhi, we still do not have a clear understanding of the immunological CoP for typhoid fever. Thus, a possible avenue to consider was the use of the typhoid CHIM and new technologies such as mass cytometry to not only assess new vaccine candidates, but also to tease apart the immunity generated following infection and attempt to correlate these responses to protection.

### *S*. Typhi (typhoid) CHIM model

**Figure 1:**
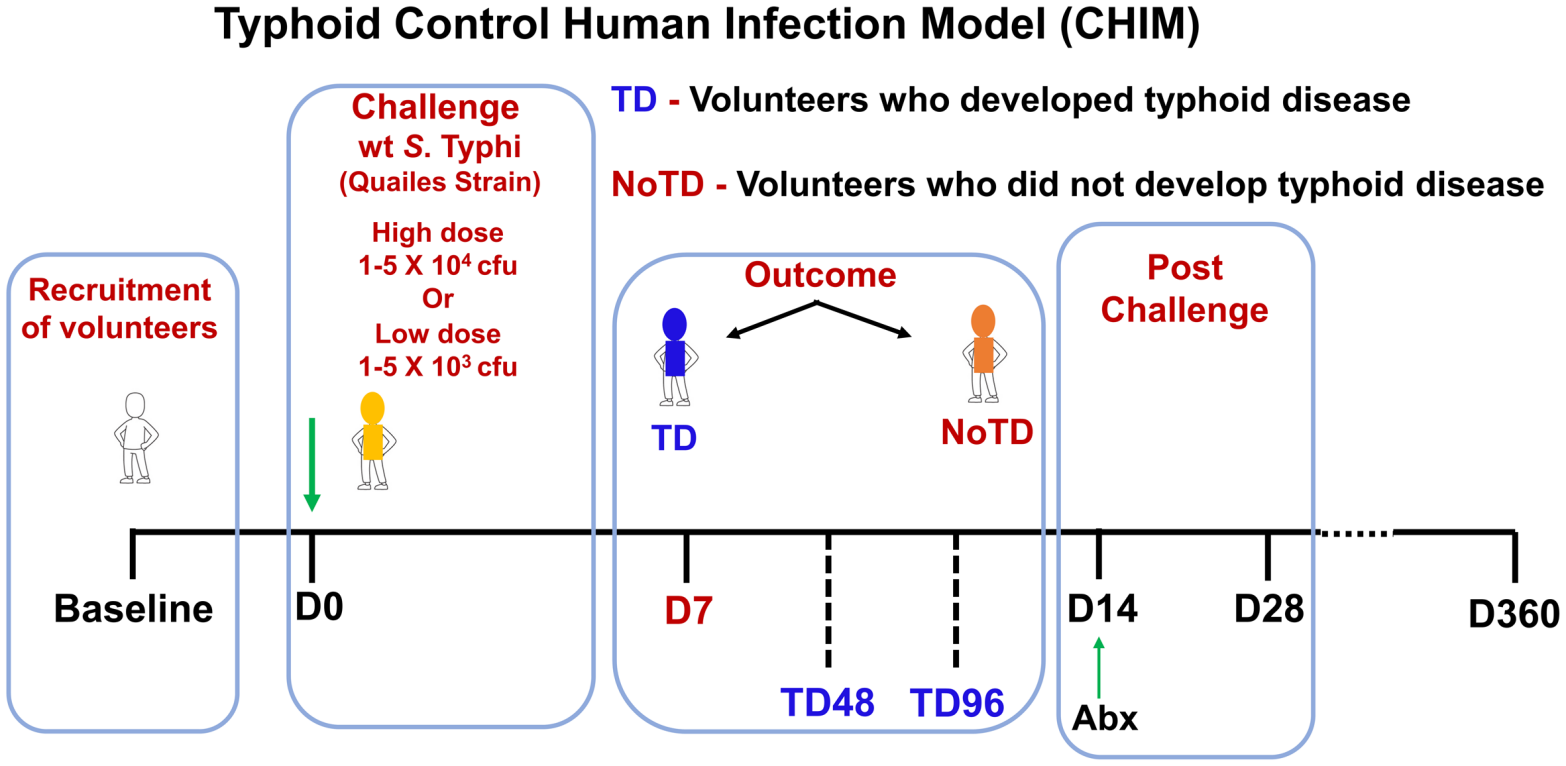
Schematic of a typhoid control human infection model (CHIM). Volunteers were recruited and challenged on day 0 with wt *S*. Typhi (Quailes Strain) at either of two doses (low—10^3^ CFU or high—10^4^ CFU). At around day 7, some of volunteers developed typhoid disease (TD) while others did not develop typhoid disease (NoTD). On day 14, all volunteers (TD and NoTD) received antibiotics (Abx). PBMC were collected from multiple time points from baseline up to 360 days after challenge.

The association between effector immune response and protective immunity is not straightforward and easy to evaluate. CHIM is an alternative and viable method for the evaluation of immunity in infectious diseases (Chakraborty et al., 2016; Chen et al., 2016). Investigation of *S*. Typhi-specific immunity, particularly CMI, has mostly been limited to studies in endemic areas or following vaccination. There are very limited data showing *S*. Typhi-specific immune status before infection with *wt S*. Typhi or on the immunological CoP. Typhoid CHIM allows for the direct examination of correlation between baseline levels of responses directed against *S*. Typhi and the development of typhoid disease. As discussed above, the CHIM for typhoid fever was developed by Drs. Theodore Woodward and Richard B. Hornick at the University of Maryland in the 1960’s and 1970’s (Hornick et al., 1970; Woodward, 1980) and recently re-established in an ambulatory setting by Dr. Pollard and his team at Oxford University (Darton et al., 2014; Waddington et al., 2014a). In the re-established typhoid CHIM, healthy consenting volunteers ingested two dose levels (10^3^ or 10^4^ colony-forming units (CFU)) of wt *S*. Typhi (Quailes strain) and were monitored for 2 weeks. Typhoid diagnosis (TD) was defined as volunteers who developed fever (temperature > 38°C sustained for >12 h and/or bacteremia). At day 14, all volunteers, including volunteers who did not meet the definition of typhoid diagnosis (NoTD) received antibiotic treatment. The attack rates in this study were 55% (10^3^ CFU) and 65% (10^4^ CFU) indicating that some of the volunteers developed typhoid disease (TD) and others did not developed disease (NoTD) following ingestion of the bacteria (Waddington et al., 2014a). PBMC were collected at multiple time points from these volunteers as shown in Figure 1, which allowed for the interrogation of these samples using sophisticated technologies to define the immunological CoP. Our group used PBMCs from these participants to perform extensive immunological studies, including the induction of regulatory T cells (T_reg_), CD8+ T_M_ and memory B cells (B_M_) responses (McArthur et al., 2015; Fresnay et al., 2016, 2017; Toapanta et al., 2016), as well as to study the effects of challenge with wt *S.* Typhi on activation of circulating dendritic cells (DC) and macrophages (Toapanta et al., 2015), and mucosal associated invariant T (MAIT) cells (Salerno-Goncalves et al., 2017, 2022) and associate these responses with clinical outcome in an effort to define immunological CoP.

#### Innate immunity

**Figure 2:**
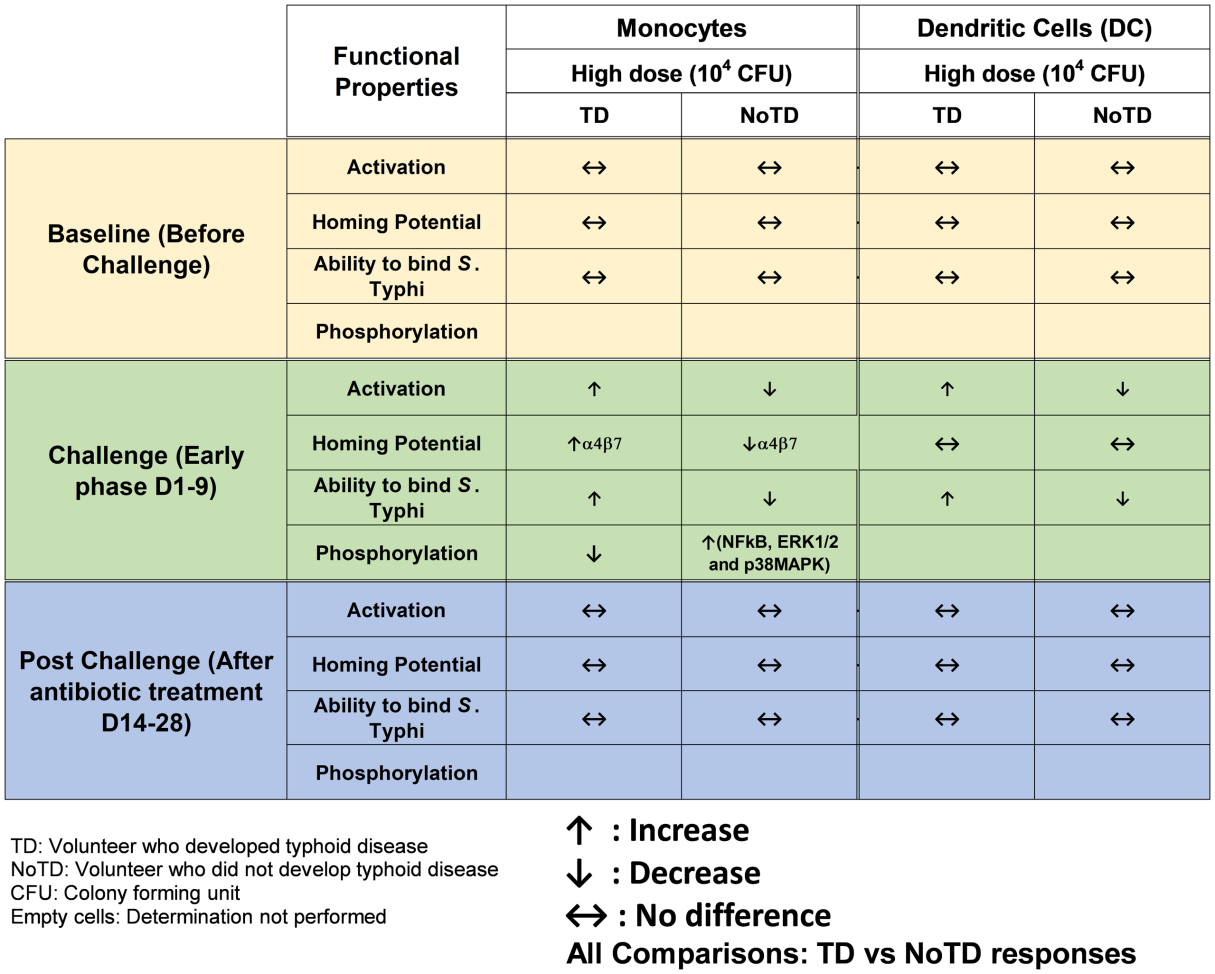
Characteristics of monocytes and dendritic cells (DC) in a typhoid CHIM. Summary of responses by monocytes and DC reported in Toapanta et al. (2015) and including comparisons between TD and NoTD at baseline (before challenge), early days after challenge (D1-9) and at later time points after challenge (D14-28). ↑: Increase; ↓: Decrease; ↔: No difference.

The innate immune system is the host’s first line of defense as these cells produced a plethora of inflammatory and antimicrobial responses which are particularly important to maintain the microbiome and pathogens at bay in the intestine. However, enteric bacteria such as *S*. Typhi have evolved to invade the intestinal mucosa and enter the submucosa where they first encounter innate cells such as dendritic cells (DC), monocytes and macrophages. Subsequently, *S*. Typhi enter the secondary lymphoid tissues [e.g., Peyer’s patches, mesenteric lymph nodes (mLN)] and finally disseminate in the systemic circulation. Most of the studies that explored the role of innate cells in *Salmonella* infection have used *S*. Typhimurium mouse models. For example, in the early phase of *S*. Typhimurium infection, the control of the bacterial growth requires reactive oxygen intermediates generated by NADPH oxidase from macrophages (Mastroeni et al., 2000). Additionally, the role of DC in the pathogenesis of *S*. Typhimurium has been found to contribute to the pathogenesis by transporting the bacteria from the intestine to mLN and other lymphoid tissues (Niess and Reinecker, 2006). Studies in mice have also shown that DC can either directly (upon uptake and processing of *Salmonella*) or indirectly (by bystander mechanisms) elicit *Salmonella*-specific CD8^+^ T-cells (Sundquist et al., 2004). Antigen presenting cells (APC) have a strategic function in the initiation and modulation of the immune responses (Segura and Amigorena, 2014) and can cross-present antigens. Among APC, DC are the most efficient *in vivo* and hence, the modulation of DC cross-presentation is important for the successful generation of strong CD8+ T cell responses to vaccine antigens. The first direct demonstration of DC function in human *S*. Typhi immunity was their ability to uptake *S*. Typhi-infected human cells and secrete IFNγ and IL-12p70 though suicide cross presentation (Salerno-Goncalves and Sztein, 2009). This process led to the presentation of bacterial antigens and the elicitation of mostly effector memory CD8+ (T_EM_) cells (Salerno-Goncalves and Sztein, 2009). Circulating monocytes have shown plasticity and the ability to differentiate into various types of macrophages and DC depending on the cytokine environment in the tissues to which they migrate. However, in humans, the mechanisms and role of these innate cells in *Salmonella* immunity is not well defined. Importantly, the role of these cells before and during infection could be very informative. Human specimens obtained immediately after infection with wt *S*. Typhi in the field is exceedingly rare. Thus, our group used PBMC obtained from volunteers (TD and NoTD) participating in the typhoid CHIM performed by the Oxford group to explore the role of DC and monocytes in *S*. Typhi infection and their potential to impact the development of typhoid disease (Toapanta et al., 2015). We studied changes in the frequency, activation, and binding ability to *S*. Typhi-infected cells, as well as signaling induced in monocytes and DC isolated from blood of TD and NoTD volunteers. Briefly, the frequencies of monocytes (CD14+ CD16+/-HLA-DR+ CD56-CD66b-CD3-CD19-) and DC (HLA-DR+ CD11c+/− CD123+/−CD14-CD3-CD19-CD56-CD66b-) were observed to be similar between TD and NoTD participants following challenge with *wt S*. Typhi, except for a small decrease in monocytes after challenge in TD volunteers. Monocytes and DC in the TD volunteers seem to be activated (upregulation in CD38 and CD40) after challenge (Figure 2). This increased activation was not detected in NoTD volunteers in either monocytes or DC. Thus, it appears that both monocytes and DC play a role in typhoid disease. The site of entry for *S*. Typhi is the intestine and hence effector cells have to selectively home to this infection site by upregulating the gut homing molecule integrin α4β7. Interestingly, this study examined the expression of integrin α4β7 on circulating monocytes and found an upregulation of integrin α4β7 in TD volunteers after challenge, but not in NoTD volunteers (Figure 2). In contrast, no changes in the expression of integrin α4β7 were observed in DC from challenged volunteers. These data suggest that monocytes and DC perform their effector functions in different target tissues. In addition, this study determined the binding/interaction of monocytes and DC to *S*. Typhi-infected cells. Remarkably, monocytes and DC from all NoTD volunteers showed an increase in their binding abilities to *S*. Typhi immediately after challenge. Furthermore, when these NoTD monocytes and DC were stimulated *in vitro* with *S*. Typhi-LPS, phosphorylation of NFkB and p38MAPK were detected. In contrast, no phosphorylation events were detected in TD monocytes after challenge (Toapanta et al., 2015). Therefore, using samples from typhoid CHIM, this study was able to demonstrate the characteristics and functions of circulating monocytes and DC throughout *S*. Typhi infection.

#### Adaptive immunity

Enteric bacteria (*S*. Typhi) engage not only innate immunity but also adaptive immunity which is complex and requires the involvement of both the humoral and cell mediated immunity (CMI) arms to protect from infection. CMI is essential in eliminating intracellular pathogens (e.g., *S.* Typhi-infected cells) while the humoral arm (antibodies) is usually associated with defense against extracellular bacteria. However, it has now been recognized that both antibodies and CMI can play complementary roles in protection against intracellular and extracellular pathogens (Kirimanjeswara et al., 2008). We and others have studied the relative contribution of serum antibodies, secretory IgA (SIgA), CD4+, CD8+, and other T-cell subsets (e.g., MAITs, T_RM_), including the interaction between T, B, and APC which together are likely to contribute to an effective immune response against typhoid fever [reviewed in Sztein et al., 2014]. These studies were performed using specimens from volunteers immunized with typhoid vaccines. However, we do not know the role played by each effector arm (humoral and cellular) throughout the infection (e.g., pre-challenge, challenge, and post-challenge). Thus, the use of CHIM is essential in deciphering these effector immune responses and understanding the correlates of protection for each pathogen.

##### B cells

B cells are critical component of adaptive immunity as their main function is to produce antibodies. However, B cells functions are more complex (e.g., antigen presenting cells and cytokine producers) and involve multiple subsets (e.g., B memory subsets) that contribute differently (Wei et al., 2007; Sanz et al., 2008; Qian et al., 2010). For example, unswitched B memory cells (CD27+ IgD+; Um) appear to play an important role in response to encapsulated pathogens (e.g., *S*. pneumonia; Kruetzmann et al., 2003, 2012; So et al., 2012). In the case of *S*. Typhi, there is evidence that the B cell compartment is important for protection, as shown by the ability of Ty21a to elicit serum IgG antibodies against lipopolysaccharide (LPS) O-antigen which in some studies correlated with the level of protection from typhoid disease (Levine et al., 2001). Interestingly, Vi capsular polysaccharides vaccines have been shown to be moderately protective against typhoid fever (Klugman et al., 1987), likely related, at least in part, to the relative inefficiency of T cell independent antigens (polysaccharides) to elicit long-term immunological memory (Weintraub, 2003). Moreover, Vi-PS is not immunogenic in young children (<2 years old; Levine et al., 2001; Hart et al., 2016). However, it has been difficult to replicate and identify the serological CoP in large vaccine efficacy trials. In a clinical study of a Vi conjugate typhoid vaccine, Thiem et al., showed that immunization elicits high levels of Vi IgG in infants (Thiem et al., 2011) while Lin et al. showed similarly that immunization with Vi-rEPA elicits equally high level of Vi IgG in toddlers (aged 2–5 years old; Lin et al., 2001). Interestingly, Kossaczka et al. evaluated the same Vi conjugate vaccine in adults and 5–14 years old children (Kossaczka et al., 1999). The authors found that higher anti-Vi IgG levels were elicited in toddlers (2–5 years old) than in adults and children (5–14 years old) following immunization with Vi-rEPA conjugate vaccines (Kossaczka et al., 1999). Similarly, our group have recently reported differences in *S*. Typhi-specific cellular immune responses following Ty21a vaccination between adults and children (Rudolph et al., 2019a,b,c). Thus, differences in the immune responses elicited in adults participating in CHIM studies and children, the target population for this vaccine in endemic countries, should be taken into consideration when assessing novel vaccine candidates.

The development of a new generation of conjugated protein-polysaccharide vaccines such as Vi-TT offers great promise to control typhoid fever through immunization. The typhoid CHIM was used to understand the relationship between antibodies to Vi and protection against typhoid disease. Dahora et al. showed that Vi-TT vaccination induces higher fold change in anti-Vi-IgA and anti-Vi IgG1 avidity in protected volunteers (NoTD) than in TD participants (Dahora et al., 2019). They also noted that while Vi-PS and Vi-TT have similar vaccine efficacies, they may protect through different mechanisms (Dahora et al., 2019). To address this further, another typhoid CHIM study was conducted to examine the cellular components involved in response to Vi-TT and Vi-PS immunization using mass cytometry and Vi-ELISPOT (Cross et al., 2020). The authors observed that Vi-specific IgG and IgM were significantly induced in Vi-TT vaccinated volunteers along with modest T follicular helper cells responses (Cross et al., 2020). Interestingly, the authors also observed a significant increase in integrin α4β7+ CCR10+ IgA+ plasma cells in both vaccine groups. The authors concluded that both gut homing and systemic antibodies may be critical for protection following Vi-TT vaccination (Cross et al., 2020). To further address the serological CoP, Jin et al. used the typhoid CHIM and a systems serology approach to identify Vi-specific serological CoP (Jin et al., 2021). Remarkably, the authors observed that protection against *S*. Typhi infection correlated with the Vi IgA quantity and avidity, while increases in Vi IgG responses were correlated with reduced disease severity. Moreover, using a systems serology approach, it appears that there is a synergy of multiple factors such as polyclonal antibody responses (avidity and innate immune cell functional responses) and Vi IgA and IgG quantity that contribute to protection against typhoid fever (Jin et al., 2021). Taken together, these data support the notion that multiple serological components, rather than a single function (e.g., neutralizing antibodies) are sufficient to prevent *S*. Typhi infection, complicating the ability to identify serological CoP. Of note, the CoP depend on the vaccine being studied. Ty21a, a Vi-negative strain, provide long-lasting protection against typhoid fever and LPS alone does not appear to correlate with protection following Ty21a immunization. Interestingly, it was only recently that our group provided the first direct evidence of *S*. Typhi-specific B_M_ cells (IgA and IgG anti-LPS and -Vi) in volunteers immunized with vaccines against *S*. Typhi (Wahid et al., 2011, 2012). Taken, together, these data suggest that not only antibody responses, but CMI mechanisms as well, are very likely to play major roles in protection against *S*. Typhi infection.

**Figure 3:**
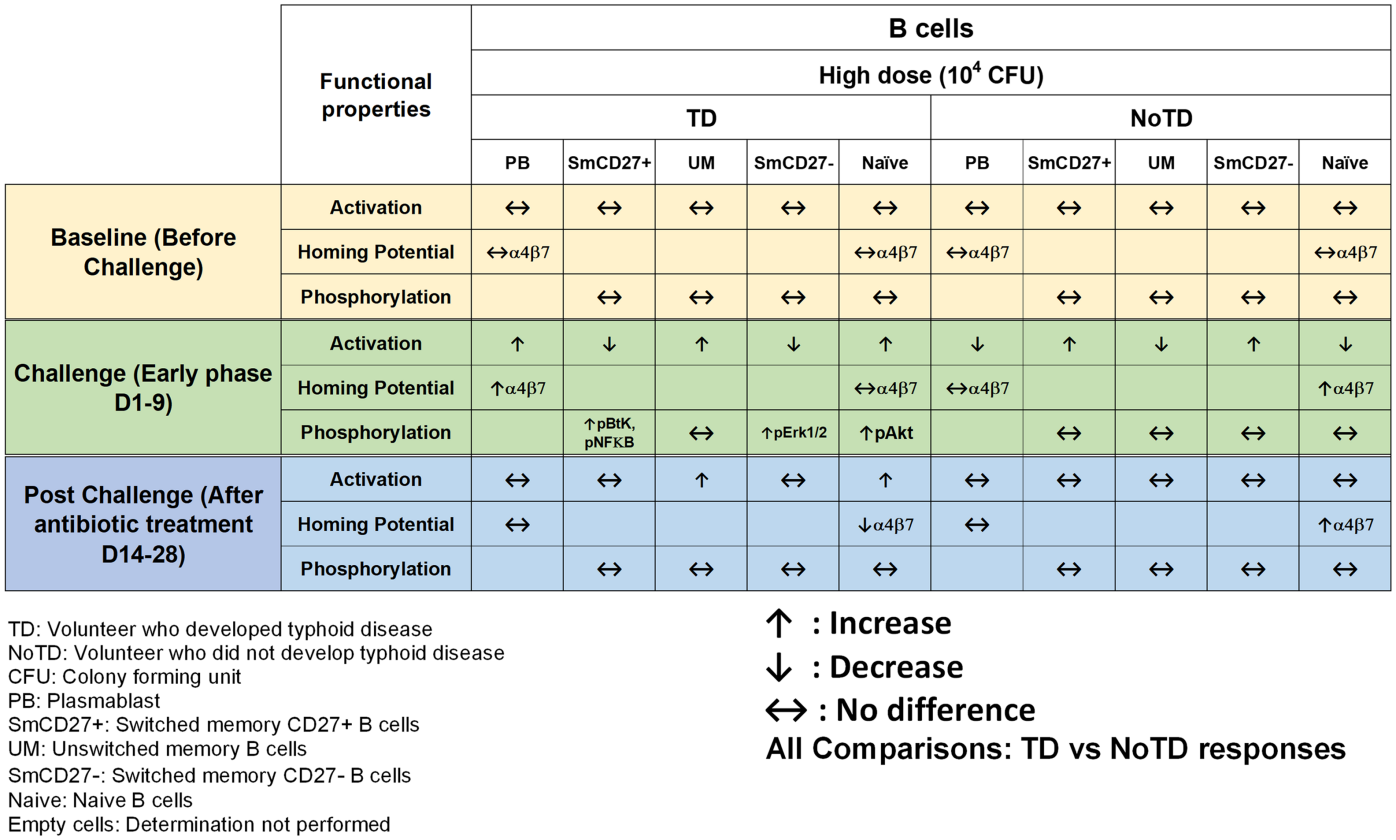
Characteristics of B cells in a typhoid CHIM. Summary of *S*. Typhi-specific B cell responses reported in Toapanta et al. (2016) including comparisons between TD and NoTD at baseline (before challenge), early days after challenge (D1-9) and at later time points after challenge (D14-28). ↑: Increase; ↓: Decrease; ↔: No difference.

Until recently, it remained unknown whether a specific B cell subset has a predominant function in typhoid disease. To address this issue, using PBMC samples from the Oxford typhoid CHIM, Toapanta et al. described the contribution of B cells subsets during *S*. Typhi infection (Toapanta et al., 2016). The authors described the changes in the frequencies, activation, and signaling in memory B subsets including plasmablasts (PB) throughout the typhoid CHIM. They observed that all participants have relatively similar frequencies of B cells, B_M_ subsets and PB before the challenge. However, following challenge with wt *S*. Typhi, B cells, switched memory (Sm, CD27+) and unswitched (Um) cells were significantly decreased in TD compared to NoTD volunteers. However, these cells were shown to be activated, as determined by upregulation of CD21 and CD40. In addition, the expression of integrin α4β7 was upregulated following *S*. Typhi challenge in TD volunteers suggesting that some of these cells had the potential to migrate to the gut, i.e., the primary site of infection. Moreover, the authors observed a positive correlation between CD21 upregulation by PB and the increase in anti-flagella antibody titers. Finally, changes in the signaling profile of particular B_M_ subsets were observed following *S*. Typhi-LPS stimulation. Interestingly, TD naïve B cells showed higher level of phosphorylating Akt than their NoTD counterparts after challenge (Figure 3). Therefore, the use of specimens from the typhoid CHIM allowed for the assessment of B cells subsets and their activation throughout the course of the disease. This study was the first to show differences in B cell subsets directly related to clinical outcome following oral challenge with wild-type *S*. Typhi in humans.

##### CD8+ T cells

**Figure 4:**
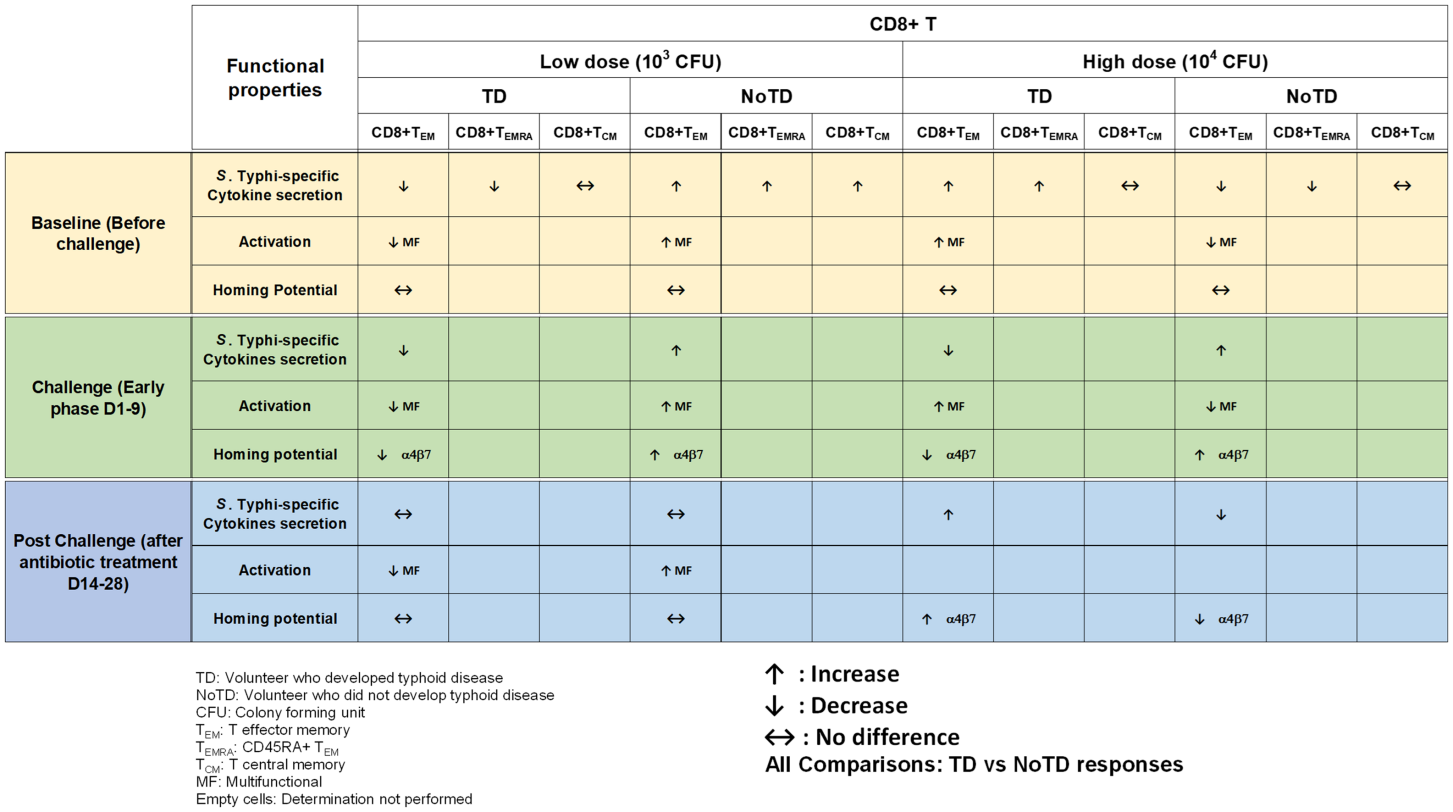
Memory CD8+ T cells responses in a typhoid CHIM. Summary of *S*. Typhi-specific CD8+ T memory cell responses reported by Fresnay et al. (2016, 2017), including comparisons between TD and NoTD at baseline (before challenge), early days after challenge (D1-9) and at later time points after challenge (D14-28) with either of two doses of wt *S*. Typhi (Low: 10^3^ CFU and high: 10^4^ CFU). ↑: Increase; ↓: Decrease; ↔: No difference.

Effector CD8+ T cells are major components of the immune responses against intracellular bacteria such as *S*. Typhi. There is considerable evidence that CD8+ T cells may play a crucial role in the control of typhoid fever (Sztein et al., 1995; Salerno-Goncalves et al., 2002; Sztein, 2018). CD8+ T cells have been shown to destroy infected-host cells through cytolytic activity and/or production of cytokines (e.g., IFN-γ, TNF-α, and IL-17; Sztein et al., 1995; Salerno-Goncalves et al., 2002; McArthur and Sztein, 2012). Recent studies in participants immunized with Ty21a have revealed that CD8+ T immune responses are mostly multiphasic and multifunctional (simultaneous production of multiple cytokines) to antigenic presentation by class Ia HLA and also by non-classical HLA-E molecules (Sztein et al., 1995; Salerno-Goncalves et al., 2004; Wahid et al., 2008; Salerno-Goncalves and Sztein, 2009; McArthur and Sztein, 2012). However, the studies mentioned above were not able to establish whether CD8+ *S*. Typhi-specific responses are associated with clinical outcome. The use of typhoid CHIM was instrumental in determining the role of CD8+ T cells in typhoid disease and whether there were correlations between CD8+ *S*. Typhi responses and clinical outcome. Fresnay et al., conducted two studies using specimens from the Oxford typhoid CHIM studies [(1) low challenge dose of *wt S*. Typhi—10^3^ CFU and (2) high dose—10^4^ CFU] to examine these questions in detail (Fresnay et al., 2016, 2017). Remarkably, the study with low dose (10^3^ CFU) of *wt S*. Typhi challenge organisms provided the first evidence that CD8+ *S*. Typhi-specific responses correlate with clinical outcome in humans (Fresnay et al., 2016). Briefly, higher level of multifunctional *S*. Typhi responsive CD8+ T cells at baseline were associated with protection against typhoid and delayed disease (Figure 4). In addition, it was observed that after challenge and following development of typhoid diagnosis, there was a decrease in circulating integrin α4β7+ *S*. Typhi-responsive CD8+ T effector memory (T_EM_). However, protection against disease was associated with low or no changes in circulating *S*. Typhi-responsive T_EM_ (Fresnay et al., 2016). Interestingly, when the authors analyzed the *S*. Typhi high challenge dose (10^4^ CFU) data, they found that higher baseline responses against *S.* Typhi correlated with typhoid diagnosis (Fresnay et al., 2017). This is in stark contrast to what was observed with the low dose (10^3^ CFU; Figure 4). The authors explained this difference by speculating that the higher number of *S*. Typhi in the high dose inoculum might have overwhelm the immune system resulting in strong inflammatory responses which could favor the systemic spread of *S*. Typhi leading to the development of typhoid fever. Importantly, higher levels of classical and non-classical (HLA-E) class I MHC-restricted *S*. Typhi baseline responses against *S*. Typhi were observed among TD volunteers. This suggests that *S*. Typhi-responsive CD8+ T cell are likely to play key roles in protection. In addition, *S.* Typhi-responsive integrin α4β7− and integrin α4β7+ CD8+ T_EM_ cells were decreased in TD volunteers after challenge (Figure 4). These data suggest firstly that migration of the CD8+ T_EM_ might be associated with the development of typhoid fever and secondly that *S.* Typhi-responsive T_EM_ cells migrate not only to mucosal sites but also to other sites. These results are in agreement with previous observations in the group of Oxford participants challenged with the lower dose of *S.* Typhi (Fresnay et al., 2016) and after vaccination (Salerno-Goncalves et al., 2005; Sztein, 2007; Wahid et al., 2008). In this stringent CHIM study, volunteers were exposed to high doses (10^4^ CFU) of the pathogen which likely exceed the number of infectious *S*. Typhi organisms that one would ingest naturally during an infection. Therefore, the use of typhoid CHIM not only allowed to uncover correlations between *S*. Typhi-responsive cells to clinical outcomes but also allowed a first look into how different doses of bacteria might influence clinical outcome.

##### Regulatory T cells

**Figure 5:**
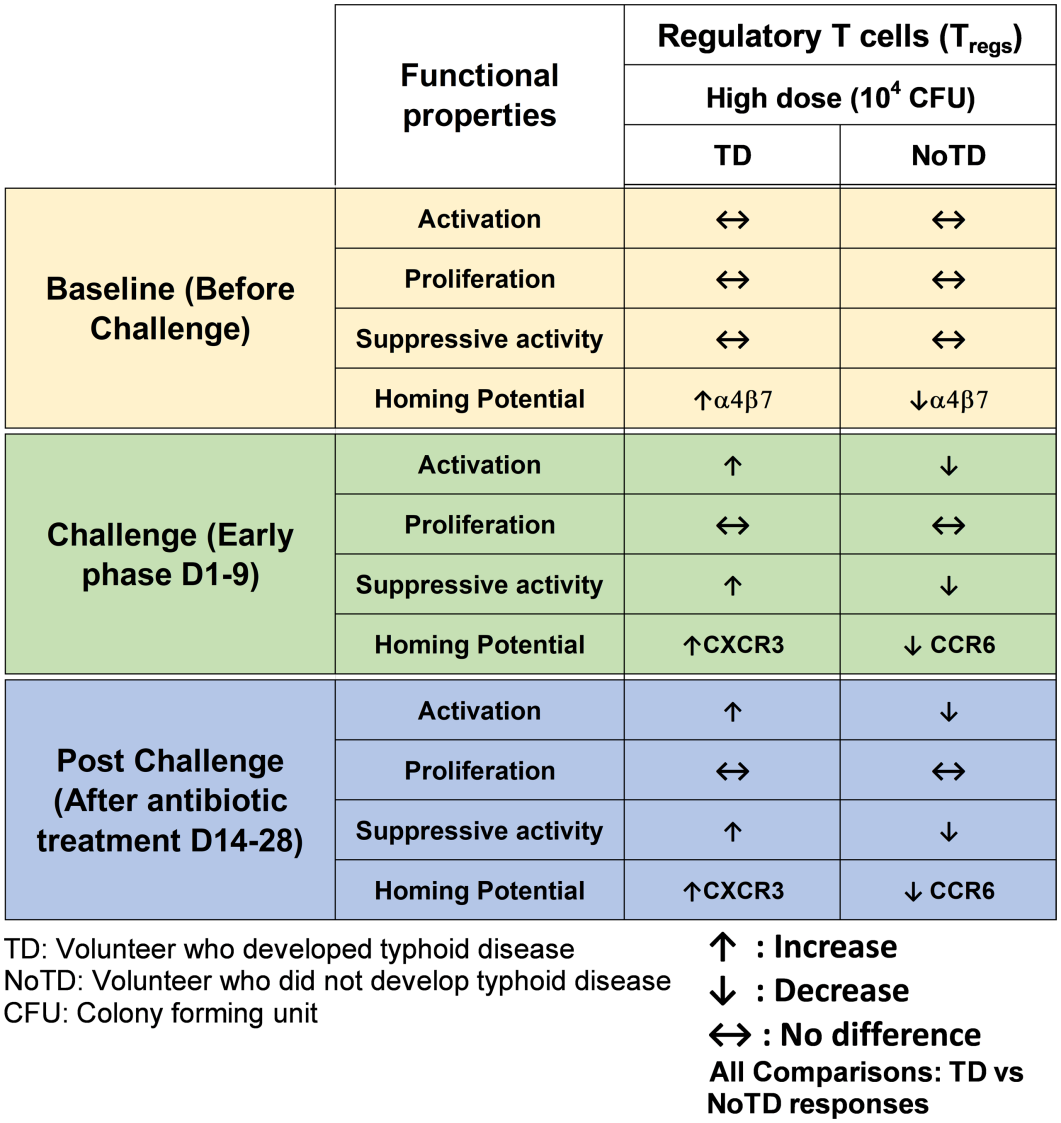
Characteristics of regulatory T cells (T_regs_) in a typhoid CHIM. Summary of *S*. Typhi-specific T_regs_ responses reported by McArthur et al. (2015) including comparisons between TD and NoTD at baseline (before challenge), early days after challenge (D1-9) and at later time points after challenge (D14-28). ↑: Increase; ↓: Decrease; ↔: No difference.

Regulatory T cells (T_regs_) are specialized subsets of CD4+ T cells that regulate other immune cells and play an essential role in immunological homeostasis (Kumar et al., 2018; Sakaguchi et al., 2020). T_regs_ are characterized by the expression of CD25 (interleukin (IL)-2 receptor α) and the transcription factor Forkhead box protein (Fox)P3 (Sakaguchi et al., 2009). In human, T_regs_ (CD4+ CD25+ CD127loFoxP3+) frequencies are less than 10% of CD4+ cells (Sakaguchi et al., 2020) and are heterogeneous. During the last couple of decades, the role of T_regs_ in infectious diseases (particularly of viral origin) and following vaccination have been reported (Lundgren et al., 2003; Aandahl et al., 2004; Cabrera et al., 2004; Raghavan et al., 2004). Two main roles of T_regs_ in infectious diseases has been described (Reviewed in (Veiga-Parga et al., 2013; Boer et al., 2015)). Firstly, they limit immune mediated pathology by inhibiting the proliferation of infected cells, regulating cells influx into lymph nodes and infected sites and favoring memory formation due to longer antigen availability and hence reducing the damage to the host. Secondly, T_regs_ reduced effector immunity and the clearance of the infection resulting in chronic pathogen persistence. However, studies reporting the role of T_regs_ in bacterial infections are limited (Johanns et al., 2010; Cook et al., 2014; Neill et al., 2014). Interestingly, the role of T_regs_ in *Salmonella* was described in a mouse model of *S*. Typhimurium where T_regs_ were shown to suppress early protective immunity and hence allowed the establishment of infection. However, a decrease in T_regs_ function at later time points allowed for the clearance of infection. Thus, *Salmonella*-specific T_regs_ might be elicited by *Salmonella* antigens and suppressed *Salmonella*-induced effector responses might contribute to the development of disease (Johanns et al., 2010). Since there are no animal models that can fully recapitulate human typhoid fever, McArthur et al. used specimens collected in the Oxford typhoid CHIM study (high dose—10^4^ CFU) to explore the role of T_regs_ in *S*. Typhi infection in human by examining the characteristics and kinetics of T_regs_ homing potential and activation (McArthur et al., 2015). Of note, the functional capacity of T_regs_ to suppress *S*. Typhi-specific T cells responses following *wt S*. Typhi challenge of healthy adult volunteers was also assessed. Interestingly, using the typhoid CHIM, it was observed that in TD volunteers, there was an upregulation of integrin α4β7 (gut homing molecule) on *S*. Typhi responsive T_regs_ pre-challenge (Figure 5). However, after *wt S*. Typhi challenge, there was a significant downregulation in *S*. Typhi responsive T_regs_ in TD volunteers. These data suggest that *S*. Typhi responsive T_regs_ actively homed to the site of infection in the gut. Furthermore, *S*. Typhi responsive T_regs_ from TD volunteers were found to be activated post-challenge as determined by upregulation of activation molecules on their surface. Remarkably, it was shown that the depletion of T_regs_ (possibly *S*. Typhi responsive T_regs_) *in vitro* leads to increases in cytokine secretion by CD8+ T_EM_ (Figure 5). It is unknown whether T_regs_ responses would be different in the CHIM with the low dose *S*. Typhi challenge (10^3^ CFU). In sum, using CHIM specimens, the authors were able to demonstrate that activated T_regs_ may play a pivotal role in typhoid fever, possibly through suppression of *S*. Typhi-responsive effector T cell responses. These studies provided valuable and new insights into the regulation of antigen-specific immune responses critical in protection against typhoid and other enteric infectious diseases.

##### MAIT cells

**Figure 6:**
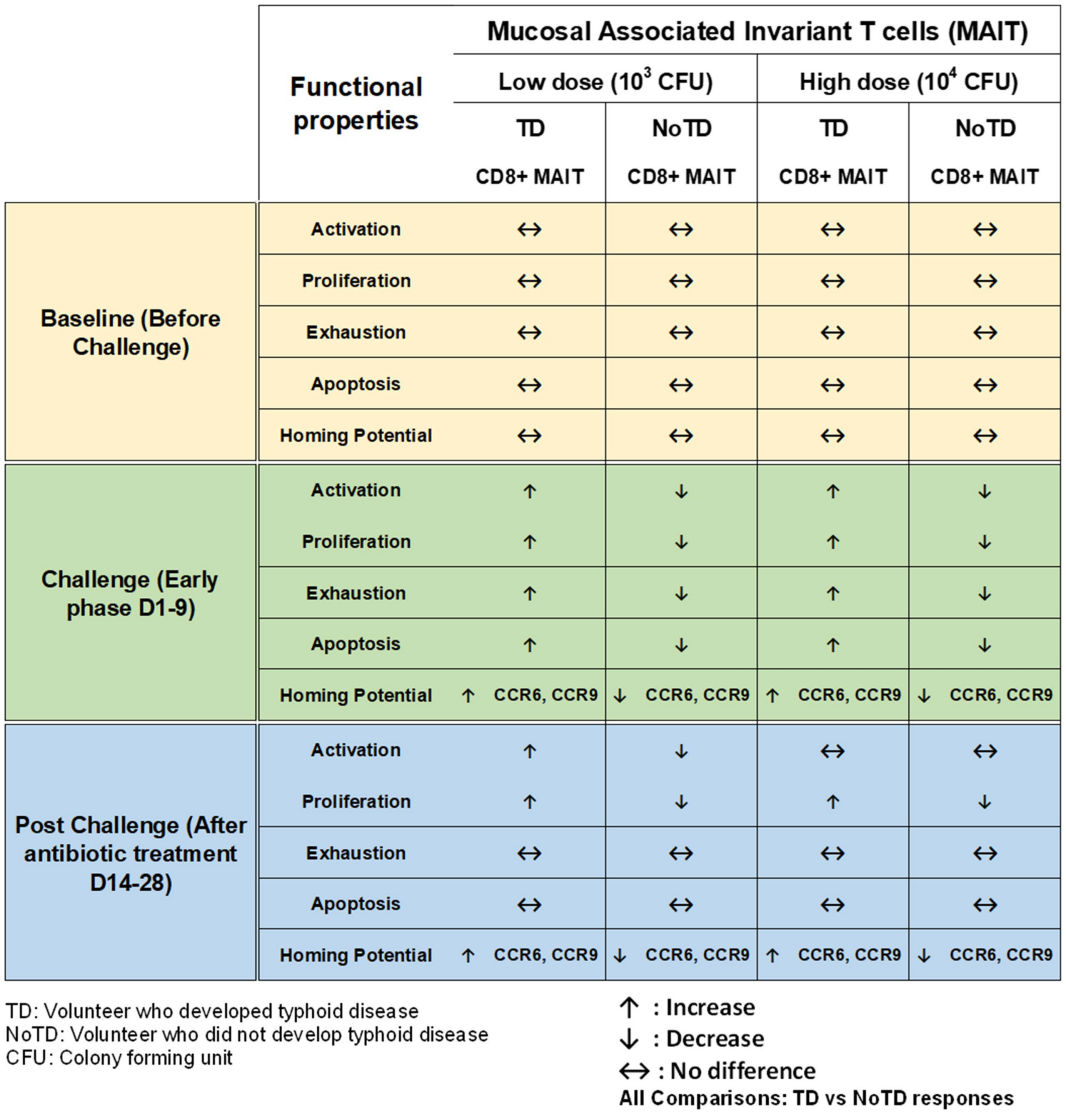
Characteristics of MAIT cells in a typhoid CHIM. Summary of MAIT cell responses reported by Salerno-Goncalves et al. (2010, 2017) including comparisons between TD and NoTD at baseline (before challenge), early days after challenge (D1-9) and at later time points after challenge (D14-28) with either of two doses of wt *S*. Typhi (Low: 10^3^ CFU and high: 10^4^ CFU). ↑: Increase; ↓: Decrease; ↔: No difference.

Mucosal associated invariant T cells (MAIT) are unconventional innate-like T cells that bridge innate and adaptive immunity and are abundant in humans. Recent studies have shown that MAITs play important roles in a broad range of infectious (bacterial and viral) and non-infectious diseases [reviewed in van Wilgenburg et al., 2016; Hinks and Zhang, 2020, and Wong et al., 2017]. For example, we have identified and characterized MAIT cells in the human gastric mucosa and examined its role in *Helicobacter pylori* infection (Booth et al., 2015). Interestingly, MAIT cells are defined by their expression of a semi-invariant αβ TCR with distinct antigen specificity for microbial riboflavin-derivatives presented by the major histocompatibility complex (MHC) class I like protein MR1. MAIT cells can exhibit rapid innate like effector responses to respond to a restricted set of ligands upon activation by TCR-dependent and independent mechanisms without expansion. Remarkably, MAIT cells have an effector/memory phenotype (CD45RA-CD45RO + CD62L-CD95 + CD44+) and are abundant in human tissues e.g., 1–4% of all T cells in peripheral blood (Booth et al., 2015), ~10% of lung T cells and 20–40% of liver T cells. We have recently shown that B cells control CD8+ MAIT cells activation and responses to *S*. Typhi, likely through regulation of the HLA-G receptor CD85j (Salerno-Goncalves et al., 2021). Furthermore, we showed that in gut MAIT cells, *S*. Typhi induced post-translational modifications in histone methylation and acetylation that are related to both gene activation and silencing (Sztein et al., 2020). However, these studies did not enable the evaluation of the existence of associations between MAIT cell responses and clinical outcome in humans. To directly explore this possibility, Salerno-Goncalves et al., used Oxford CHIM specimens to characterize human CD8+ MAIT cell immune responses to *S*. Typhi infection (both in the low- or high dose of *wt S*. Typhi cohorts) to define the kinetics of CD8^+^ MAIT cells following challenge, as well as their levels of activation, proliferation, exhaustion/apoptosis, and homing potential (Figure 6; Salerno-Goncalves et al., 2017). Interestingly, in TD volunteers, the authors observed a sharp decline in the frequencies of circulating CD8+ MAIT cells during the development of typhoid disease, regardless of the challenge dose (Salerno-Goncalves et al., 2017). In contrast, in NoTD volunteers there was little or no change in the frequencies of CD8+ MAIT cells after *S*. Typhi challenge. Furthermore, it was shown that MAIT cells of TD volunteers (low dose) show higher levels of activation as demonstrated by CD38 expression than in high dose TD volunteers during the development of typhoid disease. These activated MAIT cells also co-expressed CCR9 (a gut homing molecule), CCR6 and Ki67 (a proliferation marker). In contrast, MAIT cells obtained from NoTD volunteers regardless of the dose showed no differences in activation and homing markers (Salerno-Goncalves et al., 2017). These results indicate that TD MAIT cells are activated (CD38+) and acquire homing molecules (CCR6+, CCR9+), which also varies in function of the bacterial dose. For example, in the high dose challenge TD volunteers, MAIT cells might not be recruited as actively to the inflamed intestinal mucosa as they may have to deal with the systemic dissemination occurring with this dose of challenge. Recently, Salerno-Gonvalves et al. characterized in further detail MAIT cell function and its correlation with disease outcome using samples from an Oxford typhoid CHIM study (Salerno-Goncalves et al., 2022). They observed that MAIT cells obtained from TD and NoTD volunteers displayed distinct functional signatures which were associated with protection against typhoid fever (Salerno-Goncalves et al., 2022). These observations suggest that MAIT cytokine patterns can be predictive of typhoid fever outcome. Altogether, these data suggest that MAIT cells play an integral role in immunity against *S*. Typhi and might contribute to maintain a balance between health and disease in the gut microenvironment. Future CHIM studies are needed to examine the effects of the inoculum size on MAIT cell homing behavior.

### Other *Salmonella* CHIM studies

#### *Salmonella* Paratyphi A

Reduction of the global burden of enteric fevers require not only the development of safe and effective vaccines, but also improvements in water quality, sanitation and hygiene measures. Although *S*. Typhi is the main etiological agent of enteric fevers globally, other *Salmonella enterica* serovars (e.g., *S.* Paratyphi A, B, C), which have a similar clinical presentation as *S.* Typhi, are responsible for a substantial proportion of enteric fever cases, estimated at 3.4–5.4 million infections globally per year (Crump et al., 2004). In endemic areas such as South and Southeast Asia, the annual incidence rates are estimated to be around 150 cases per 100,000 persons yearly. Additionally, both endemic and travel associated cases of *S*. Paratyphi A strains have been found to be resistant to multiple antibiotics, thereby limiting treatment options (Teh et al., 2014). The immunological CoP from *S*. Paratyphi A are unknown, and no licensed vaccines are currently available. There is evidence that Ty21a can elicit cross reactive humoral and CMI immune responses to *S*. Paratyphi A/B *in vitro* (Wahid et al., 2012) and only limited evidence from field trials point to cross-protection against *S*. Paratyphi B (Levine et al., 1987). Recently, our group evaluated the cross-reactivity of *S*. Typhi-specific T memory cells to *S*. Paratyphi A, *S*. Paratyphi B and invasive non-typhoidal *S*. Typhimurium using the typhoid CHIM (Rapaka et al., 2020). We observed that *S*. Typhi challenge elicited multifunctional CD4+ Th_1_ and CD8+ T_EM_ cells that were cross-reactive to *S*. Paratyphi A, B and *S*. Typhimurium. These data showed that *S*. Typhi induces cross-reactive multifunctional effector memory CD4+ and CD8+ T cell responses.

Because of the great need to develop vaccines against *S*. Paratyphi, a human *S*. Paratyphi A CHIM has been recently developed by Dobinson et al. to evaluate vaccines against paratyphoid fevers (Dobinson et al., 2017). In this CHIM, the authors reported an attack rate of 60% (12/20 volunteers) following challenge with a 1–5 × 10^3^ CFU dose. Interestingly, this paratyphoid challenge model had higher rates of subclinical bacteremia (55%) than the typhoid challenge model, which persisted for up to 96 hrs after antibiotic treatment (Dobinson et al., 2017). This *S*. Paratyphi A CHIM was well tolerated, had an acceptable safety profile and was therefore deemed suitable for assessment of vaccine efficacy. Interestingly, a recent study by Napolitani et al., which used PBMC samples from both the typhoid CHIM and the *S*. Paratyphi A CHIM, examined cross-reactive *Salmonella*-specific CD4+ T cells against distinct *Salmonella* serovars using various technologies including mass cytometry (Napolitani et al., 2018). They identified immune-dominant, serovar specific and cross-reactive antigens expressed during typhoid disease that represent major targets of CD4+ T cell responses to *Salmonella*. These results highlight the importance of dissecting effector T cell responses that are serovar specific and cross-reactive to the infection which could contribute to the development of vaccines capable of eliciting a wide array of CMI responses.

Vaccine efficacy trials and in-depth studies directed to investigate the immunological CoP following infection with wt *S.* Paratyphi A are ongoing with the use of high dimensional mass cytometry and other advanced immunological tools.

#### Immunological responses and cross-protection following challenge and re-challenge with *S.* Typhi and *S.* Paratypi A

*Salmonella* Typhi and Paratyphi A/B can both infect individuals and cause enteric fever. These serovars circulate in the same environment and thus have the opportunity to cause multiple infections or co-infection in endemic areas. It is unclear whether prior exposure confers incomplete protection against subsequent re-infection by the same (homologous) or different (heterologous) serovars. Interestingly, studies have proposed that protection against future clinical typhoid disease necessitate at least 3–5 episodes of typhoid exposure (Pitzer et al., 2014). In addition, it has been shown that previous typhoid fever confers moderate (~30%) protection against successive disease in human challenge model (Dupont et al., 1971). The protection and immunity generated upon paratyphoid re-infection remains unknown. Likewise, information is lacking on whether there is protection from heterologous re-infection with *Salmonella* serovars Typhi and Paratyphi. Gibani et al. examined this key question by performing a challenge/re-challenge study to identify protection from infection to homologous and heterologous *S*. Typhi or Paratyphi A infection (Gibani et al., 2020). Interestingly, the authors found that the attack rate in volunteers who underwent homologous rechallenge with *S*. Typhi or *S*. Paratyphi A was reduced compared with challenged naïve controls. A *post hoc* analysis showed that previous exposure results in about 36 and 57% reduced risk of typhoid or paratyphoid disease, respectively, on re-challenge. Remarkably, the authors observed that NoTD volunteers (those who did not meet the definition of enteric disease) on primary exposure were significantly more likely to be protected on re-challenge than TD volunteers (those who developed disease) on primary exposure. Importantly, re-infection with *S*. Typhi or *S*. Paratyphi in heterologous re-challenges did not correlate with reduced attack rate following challenge. In addition, the baseline O-, H- and Vi-antigens antibodies were compared between naïve and rechallenged volunteers. No differences were noted between TD and NoTD in antibody levels. Furthermore, no significant anti-Vi or anti-Hd IgG responses were found following *S*. Typhi challenge. Taken together, these data indicate that multiple infections can happen in endemic regions where the transmission is high and that a reduction of typhoid or paratyphoid disease can be expected upon a subsequent exposure to the same serovar. In contrast, data from the heterologous re-challenges with *S.* Typhi or *S.* Paratyphi showing a lack of correlation with reduced disease indicates that cross-protection between these serovars is unlikely. Additionally, the observations that anti-LPS antigens were not correlated to protection in this CHIM suggests that CMI is a major player in protection against *S*. Typhi and *S.* Paratyphi A following natural infection. Thus, there is a great need for vaccines that can be cross-protective to reduce the burden of enteric fevers. These CHIM models are also likely to contribute significantly to our understanding of infection-derived immunity to *S*. Typhi and *S*. Paratyphi A by identifying immunological CoP.

#### Shedding of bacteria following infection and re-infection with *S.* Typhi and *S.* Paratyphi A

One of the prerequisites for the transmission of enteric fevers is the shedding of *S*. Typhi or *S*. Paratyphi A in the stools or urine that will ultimately lead to contamination of food and/or water. Thus, vaccines that can not only induce protective immunity against enteric fevers but also reduce significantly *Salmonella* shedding in the environment, would be of great benefit. In the Maryland human challenge studies, Ty21a vaccination led to the reduction in *Salmonella* shedding in the stool (Gilman et al., 1977). However, there is limited data on the effect of other vaccines (e.g., Vi-TT) on stool shedding. Interestingly, while shedding was most common in TD volunteers (those who develop typhoid disease), NoTD volunteers (those who do not develop typhoid disease) also shed bacteria continuously for several weeks following challenge (Gibani et al., 2019). The *S*. Typhi and *S.* Paratyphi A stool shedding patterns are not well characterized. Additionally, the effect of previous *wt S*. Typhi infection on stool shedding after successive *S*. Typhi re-infections has not been examined previously. This is a question that will be very difficult to explore in field trials. However, CHIMs are uniquely well suited to define bacterial dynamics in clinical and subclinical typhoid and paratyphoid infections as volunteers are monitored closely under well-controlled conditions. Gibani et al., explored this important issue by studying the impact of vaccination on stool shedding of *S*. Typhi and *S*. Paratyphi in 6 CHIM studies (Gibani et al., 2019). The authors reported that volunteers who received either Vi-PS or Vi-TT vaccines shed less bacteria than unvaccinated volunteers. Remarkably, volunteers that were infected with *S*. Typhi in a previous CHIM study shed less than unexposed volunteers following challenge. Of interest, these studies showed that *S*. Typhi challenged participants shed more frequently than *S*. Paratyphi A exposed healthy volunteers. Thus, in these CHIM studies, it appears that natural infection or prior immunization with vaccines such as Vi-TT reduce the transmission of *Salmonella* strains.

#### Lessons learned

The development of new and improved vaccines along the traditional phases of safety, immunogenicity and clinical efficacy pipeline are usually very time consuming and costly. CHIM studies that are well planned and carefully executed can contribute enormously to provide insights for our understanding of host-pathogen interactions, identify the immunological correlates of protection, and determine host factors contributing to infection (Bambery et al., 2016). This can lead to an accelerated development and testing of vaccines by providing data on vaccine efficacy, and protection against a particular strain in a limited number of volunteers (Sekhar and Kang, 2020). Thus, CHIM studies can allow for up and down-selection and identification of promising vaccine candidates which can then be tested and validated in field studies. The availability of CHIMs has great potential to significantly reduce the time and cost for the streaming of vaccines to the field.

One of the striking and important findings from CHIM studies is that the baseline antigen-specific responses are important as they can correlate with clinical outcomes. The presence of preimmunization/pre-challenge immunity in humans and its impact on vaccination and infection has not been studied extensively until now. For example, in the Oxford typhoid CHIM model, higher multifunctional *S.* Typhi-specific CD8^+^ baseline responses were associated with protection against typhoid and delayed disease onset (Figure 4; Fresnay et al., 2016). In another example, baseline up-regulation of the gut homing molecule integrin α4β7 in T_regs_ was associated with the development of TD (Figure 5; McArthur et al., 2015). In contrast, baseline activation status, homing potential and frequencies of MAIT cells, a subset of CD8+ T cells, did not seem to contribute to clinical outcome for typhoid disease (Figure 6) regardless of challenge dose. We also noted that baseline frequencies, activation status and homing potential of monocytes, DC and B cells do not appear to correlate with typhoid disease clinical outcome (Figures 2, 3). These results suggest that multifunctional *S.* Typhi-specific CD8^+^ baseline responses are important and that they have the capacity to home to the site of infection in the intestine. Higher baseline T_regs_ expressing the homing molecule integrin α4β7 may suppress the multifunctional *S.* Typhi-specific CD8^+^ responses and hence provide a welcoming environment for *S.* Typhi resulting in the development of typhoid disease. Thus, we learned not only that effector and memory CD8^+^ responses appear to be a dominant effector mechanism of protection from natural *S*. Typhi infection (and perhaps also *S*. Paratyphi), but also that T_regs_ appear to also contribute to disease, suggesting that the simultaneous evaluation of both responses might be important in determining clinical outcome. Importantly, these results from the CHIM studies clearly indicate that baseline responses must be taken into consideration during the development and evaluation of novel vaccines.

Another interesting observation and lesson learned from these CHIM studies is that after challenge with the pathogen (e.g., *S.* Typhi), it seems that those volunteers who develop typhoid disease (TD) exhibit activated DC and monocytes which are nevertheless defective in defined signaling pathways (Figure 2). This suggest that these APC might be highjacked by the pathogen due to the high dose of the pathogen. Remarkably, B cell subsets are induced differentially in TD vs. NoTD volunteers (Figure 3). For example, after challenge, plasmablasts, unswitched memory (UM) and naïve B cells are activated in TD but not in NoTD volunteers. The role that these different B subsets play in contributing to typhoid disease need to be investigated in further detail. MAIT cells (at both challenge doses) were also more activated in TD than in NoTD volunteers after challenge (Figure 6). Although MAIT cells are activated and proliferate, they seem to undergo apoptosis and be exhausted in TD volunteers (Figure 6) and perhaps unable to contribute to the clearance of the pathogen. Of note, very recently we reported in participants challenged with wt *S.* Typhi an association between defined MAIT cell cytokine patterns and typhoid fever clinical outcome. These results add additional support to the notion that MAIT cell responses are important in typhoid fever. In sum, after challenge, in TD volunteers, there are several cell types that are involved in the response but in some individuals are unable to clear the pathogen, leading to typhoid disease. It is possible that part of the problem is that these cells are not optimally activated and/or may not home efficiently to the site of infection in the intestine to appropriately combat the pathogen in the local microenvironment. On the other hand, it is also possible that these varying degrees of activation on various cell lineages represent the stimulation of these effector cells following extended exposure to the pathogen, a phenomenon that is not present in NoTD volunteers.

Finally, it is important to keep in mind that PBMC samples represent a window in time for the assessment of immune responses. The lack of responses in circulation at defined times may be interpreted as a consequence of circulating antigen-specific cells that might have migrated to the appropriate microenvironment (the gut and secondary lymphoid tissues in the case of *S.* Typhi) to exert their effector function. Therefore, it is crucial that blood specimens be obtained at different time points to define the kinetics of the responses and ensure that the immunity elicited is captured in circulation. Alternatively, whenever possible, biopsy samples of the site of infection (terminal ileum for *S.* Typhi) could be obtained and immune responses assessed.

In sum, the use of human challenge studies holds great potential to dramatically accelerate our understanding of the complex immune responses that ensue following exposure to wt *Salmonella*. Together with the advent of extremely powerful technologies such as single-cell transcriptomics and mass cytometry, as well as the use of systems biology approaches, there is great potential to acquire critical information to dramatically accelerate the rational design of long-term protective vaccines.

#### CHIM limitations and pitfalls

As described throughout this review, the recent interest in CHIM studies has been driven by their unique ability to contribute to vaccine development and efficacy and our understanding of immune responses to infections, including the identification of CoP. However, as with any models, CHIMs are not without their limitations. Among these are the following: (i) it is important to recognize that CHIM studies have strict inclusion and exclusion criteria. The results may not always be fully generalizable and careful consideration is needed before extrapolating data obtained from these studies (Sekhar and Kang, 2020); (ii) Healthy adults (20–30 years old) from developed countries are usually enrolled in these studies and thus may not represent the population at risk which usually involves children residing in developing countries (Shirley and McArthur, 2011); (iii) There are many factors that differ between CHIM volunteers and the population at risk which include genetic background, age, nutrition, microbiome/microbiota, co-morbidities, co-infections, background immunity and challenge or exposed doses of pathogen. CHIM volunteers tend to be a naïve population that has never been exposed to the pathogen whereas the population at risk in endemic regions is very likely to have been previously exposed to the pathogen or a closely related pathogen. CHIMs are not usually carried out in Low- and Middle-Income Countries (LMIC) because of various factors including technical, clinical, ethical and regulatory issues, as well as cultural norms. However, it would be greatly beneficial to conduct these studies in LMIC settings than high income countries as there are different host-pathogen relationships in LMIC for enteric and other diseases (Kapulu et al., 2018); (iv) The route of infection and inoculum dose of challenge agent may not mimic natural infection (Roestenberg et al., 2018); (v) The challenge strain in CHIMs may be different from circulating strains and is chosen for stability; (vi) CHIM studies are often designed to evaluate short term protection; however long-term protection is more desirable. Despite these limitations, CHIMs remain important models that will contribute to our understanding of human diseases and allow for the development and testing of vaccine candidates.

## Overall conclusion

Controlled human infection models (CHIM) represent a path forward in the identification of immunological correlates of protection, and to more fully understand disease pathogenesis for enteric and other infectious organisms in humans exposed to wt organisms. Moreover, CHIM studies hold great promise to accelerate the development and assessment of protective efficacy of novel vaccines.

## Author contributions

MS and JB contributed to the writing of the manuscript and read and approved the final and submitted version.

## Funding

This work was supported, in part, by NIAID, NIH, DHHS federal research grants R01 AI036525, U19 AI082655 [Cooperative Center for Human Immunology (CCHI)], U19-AI109776 [Center of Excellence for Translational Research (CETR)], and U19-AI142725 to MS. JB was supported, in part, by the Infectious Diseases Clinical Research Consortium (IDCRC) through the NIAID of the NIH, under award number UM1AI148684. The content is solely the responsibility of the authors and does not necessarily represent the official views of the National Institutes of Health.

## Conflict of interest

The authors declare that the research was conducted in the absence of any commercial or financial relationships that could be construed as a potential conflict of interest.

## Publisher’s note

All claims expressed in this article are solely those of the authors and do not necessarily represent those of their affiliated organizations, or those of the publisher, the editors and the reviewers. Any product that may be evaluated in this article, or claim that may be made by its manufacturer, is not guaranteed or endorsed by the publisher.
